# Working memory performance in the elderly relates to theta-alpha oscillations and is predicted by parahippocampal and striatal integrity

**DOI:** 10.1038/s41598-018-36793-3

**Published:** 2019-01-24

**Authors:** Tineke K. Steiger, Nora A. Herweg, Mareike M. Menz, Nico Bunzeck

**Affiliations:** 1 Institute of Psychology I, University of Luebeck, 23562 Luebeck, Germany; 2 Department of Systems Neuroscience, University Medical Center Hamburg-Eppendorf, 20246 Hamburg, Germany; 3 Department of Psychology, University of Pennsylvania, Philadelphia, PA 19104 USA

## Abstract

The ability to maintain information for a short period of time (i.e. working memory, WM) tends to decrease across the life span with large inter-individual variability; the underlying neuronal bases, however, remain unclear. To address this issue, we used a multimodal imaging approach (voxel-based morphometry, diffusion-tensor imaging, electroencephalography) to test the contribution of brain structures and neural oscillations in an elderly population. Thirty-one healthy elderly participants performed a change-detection task with different load conditions. As expected, accuracy decreased with increasing WM load, reflected by power modulations in the theta-alpha band (5–12 Hz). Importantly, these power changes were directly related to the tract strength between parahippocampus and parietal cortex. Furthermore, between-subject variance in gray matter volume of the parahippocampus and dorsal striatum predicted WM accuracy. Together, our findings provide new evidence that WM performance critically depends on parahippocampal and striatal integrity, while theta-alpha oscillations may provide a mechanism to bind the nodes within the WM network.

## Introduction

Working memory (WM) can be defined as the ability to process and maintain information for a short period of time. Performance in WM tasks typically declines with advancing age and, at the same time, shows increased inter-individual variability. More specifically, some elderly experience a considerable decline but others show well-preserved WM functioning that operates at a level of healthy young counterparts^1^. Although previous studies have identified brain changes that can explain differences between young and old subjects, the precise neural bases underlying the inter-individual differences in WM performance in the elderly population itself still remain unclear.

In general, WM is the result of several cognitive processes. During a visual WM task, these include (a) selective attention to perceptual and related long-term memory object representations during the encoding of information, (b) sustained attention operating on the previously given perceptual information and related long-term memory representations as well as rehearsal processes during retention, and (c) selective attention and pattern completion at the retrieval stage^2^. Accordingly, WM might not rely on a single brain area, but on a coordinated network, including dorsolateral prefrontal cortex (dlPFC)^3,4^, parietal cortex^5,6^, medial temporal lobe (MTL)^7,8^ and basal ganglia^9,10^.

The retention phase of a visual WM task, in which the stimulus to-be-remembered is physically absent, has been of special interest in several studies using magneto- or electroencephalography (M/EEG). Neuronal activity during retention has often been linked to changes in the theta (4–7 Hz) and alpha (8–14 Hz) band^11–14^. Furthermore, these frequency bands are both modulated by WM load (i.e. the number of items that had to be maintained). Interestingly, whereas some studies found an increase in theta and alpha power with increasing load^15,16^, others report the opposite pattern^11^ or differential effects, e.g. a decrease in theta and lower alpha band (5–9 Hz), but an increase in higher alpha (10–14 Hz)^12^. Notably, aging studies indicate that these WM related modulations might change with age^17–19^, but empirical data remain scarce.

Interestingly, WM performance is not only associated with activity in the dlPFC, parietal cortex, basal ganglia and MTL^5,7,10,20^, but also their structural integrity^21–23^ and connectivity^24^. For instance, Charlton and colleagues found associations between WM and white matter damage in temporo-frontal, temporo-parietal and fronto-parietal tracts in healthy elderly subjects^24^. Further evidence comes from lesion studies reporting a direct relationship between PFC or basal ganglia integrity and WM performance^21,22^. Importantly, all these areas and their interconnections may change during healthy aging^25–28^ further indicating a direct relationship to WM performance.

Here, we aimed to identify the underlying mechanisms of inter-individual variability in healthy elderly humans, using a multimodal imaging approach assessing cortical grey matter (VBM), subcortical white matter tracts (DTI) and neural oscillations (EEG) during a WM task. We hypothesized that WM performance closely relates to theta-alpha oscillations, and that inter-individual variability can be explained by structural integrity of the dlPFC, parietal cortex, basal ganglia and MTL as well as fronto-parietal, temporo-parietal and temporo-frontal trajectories. Furthermore, we expected that structural connectivity between critical notes of the WM network relates to theta and alpha oscillations. We tested these hypotheses within a population of healthy elderly adults for two reasons. First, while WM tends to declines with age, it also shows large inter-individual variability with rather low performance in some elderly but “youth-like” performance in others^1^. Second, a structural decline of areas important for WM functioning is common in healthy aging^25–28^. A relation between structure, neuronal activation and behavior seems plausible and was therefore the main target of investigation. A similar approach has already been used in previous studies^24,29–31^.

## Material and Methods

### Participants

A group of 32 healthy elderly participants was tested; one participant had to be excluded due to brain anomalies. The remaining 31 participants were all right-handed with normal or corrected-to-normal vision and color-vision, and none of them reported a history of neurological or psychiatric disorders or current medical problems, apart from high blood pressure (14 males; mean age = 67.3 years, S.D. = 6.2 age-range 56–78).

A wide age range (56–78 years) in our sample of elderly subjects was chosen for two reasons: First, structural and associated cognitive changes have been reported with an onset of around 55 years^25,32,33^ and previous studies are based on a similar age range^29,31,34^. Second, a wider age range offers more variance in the data (brain structure and behavior), which is necessary in order to compute correlation analyses. Note that there were no outliers in our sample of elderly subjects regarding age and brain structure.

To assess mental well-being and cognitive integrity, all participants completed the Geriatric Depression Scale (mean GDS = 1.4, S.D. = 1.9, GDS ≤ 5 for all participants; GDS ranges from 0–15, scores higher than 10 indicate depression)^35^ and the neuropsychological battery of the “Consortium to Establish a Registry for Alzheimer Disease” (CERAD)^36^ including the Mini Mental State Examination (MMSE; mean MMSE = 29.5, S.D. = 0.77, MMSE ≥ 27 for all participants; MMSE ranges from 0–30, scores smaller than 25 indicate pathologies^37^). Additional data from this group of elderly participants have been published previously^31,38^. The study was approved by the local ethics committee (Medical Association Hamburg) and all research was performed in accordance with the relevant guidelines and regulations. Each participant gave informed written consent prior to testing and received a reimbursement of 10 Euro/hour.

### Experimental design

**Figure 1:**
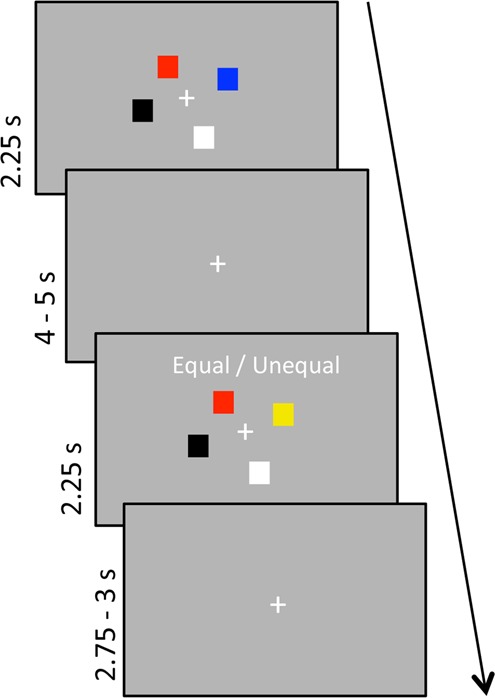
WM task design. Participants had to retain the colors of two, four or six squares (sample array) and indicate if one of the squares had changed color or not in the test array.

To measure WM performance, we employed a change-detection-task (Fig. 1). Here, an array of two, four or six colored squares was presented on a grey background for 2250 ms (sample array), followed by a jittered retention interval of 4000–5000 ms. The subsequent test array was equal to the sample array in half of the trials (no-change trials). On the other half of trials, one randomly selected square changed its color (change trials). The test array was displayed for 2250 ms, with the prompt “Equal/Unequal” and participants indicated the status of the array by button press using index and middle finger of the right hand. In the inter-stimulus interval, a fixation cross was presented for 2750–3000 ms. All colors were highly discriminative and randomly assigned to a square position (yellow, pink, green, white, red, blue, black). Each load condition (two, four or six squares, referred to as low, medium and high load, respectively) was presented 60 times, resulting in 180 trials in total. The experiment was divided into three sessions (60 trials each; 20 per condition) with five-minute breaks in-between. WM load conditions were presented randomly intermixed in each session.

### Statistical analyses of behavioral data

All data was analyzed using MATLAB (version 2014b; The Math-Works Inc.) or MATLAB-based toolboxes, further specified in the relevant sections. The individual median reaction times (RT) were calculated for correct change/stay detections only. As a measure of accuracy, *d’* was calculated in accordance with the signal detection theory^39^. Briefly, trials containing a change of color in one square were defined as hits (H), if identified correctly, and as misses (M), if the change was not detected. Complementary, correct and incorrect classifications of trials with identical sample and test arrays were defined as correct rejections (CR), or false alarms (FA), respectively.

*d’* was calculated as the difference between the inverse phi of the hit rates (HR = [NH + 0.5]/[NH + NM + 1]) and false alarm rates (FAR = [NFA + 0.5]/[NFA + NCR + 1]). The inverse phi transforms HR and FAR (probabilities) into the associated z-scores. Subjects with RT or *d’* values that differ more than three standard deviations from the mean would have been excluded from further analyses – however, no such outliers were detected. The effect of memory load on RT and *d’* was analyzed using one-way repeated measures analyses of variance (ANOVAs) with the factor load (low, medium and high) using IBM SPSS Statistics (Version 21). Planned *t*-tests between load conditions (low vs. medium, low vs. high, and medium vs. high) were considered significant at a Bonferroni corrected threshold of p = 0.05/3 = 0.017.

### EEG-recording and preprocessing

Throughout the whole experiment, electroencephalographic (EEG) activity was acquired with a 60-channel system (Brain Products GmbH, Munich, Germany) and Brain Vision Recorder (Version 1.03.0003). The active electrodes (impedance levels below 20 kΩ) were positioned according to the 10–20 system and FCz was used as a reference during recording while the right mastoid served as a ground electrode. Two pairs of additional electrodes were used to control for horizontal and vertical eye movements. A sampling rate of 500 Hz and a high- (0.1 Hz) and low-pass (1000 Hz) filter were used during recording.

EEG data were preprocessed using EEGLab (version 13_4_4b)^40^. First, continuous data were high-pass (1 Hz) and low-pass (60 Hz) filtered. Subsequently, data were epoched from 725 ms before the onset of the sample array to 4000 ms after onset of the retention and down-sampled to 250 Hz.

Next, all epochs were visually inspected to identify and reject epochs containing major atypical artifacts (mostly muscle artifacts) and bad channels. Variance related to blinks and eye movements was removed using independent component analysis (ICA)^40^. Bad channels were interpolated in six participants (one single channel each). The epochs were subsequently checked with a second visual inspection and rejected when still containing artifacts. Finally, all channels were re-referenced to the average signal of all channels. After preprocessing, an average number of 41 out of 60 trials per participant and condition remained (43 for low load, 43 for medium load, and 37 for high load).

### EEG statistical analyses

The statistical analyses of the EEG data was conducted using Fieldtrip (version 2015-11-12)^41^. Time-frequency decomposition was conducted from 4 to 30 Hz using convolution of the single-trial time series with complex Morlet wavelets (4 cycles) in steps of 0.5 Hz in the frequency and 8 ms in the time domain. Power was averaged across trials for each condition of interest. Subsequently, a condition-specific relative baseline correction was applied (150-50 ms before sample array onset). Note that all conditions were presented randomly intermixed, implying no differences in baseline-activity; indeed, this assumption was further supported by non-significant cluster-based permutation tests between baselines.

For analyzing power differences between conditions during the retention phase, non-parametric cluster-based permutation tests^42^ were conducted on all scalp electrodes (60) in a time window from 1–3 s after retention onset to avoid edge effects or transient responses from visual stimuli-onset/-offset, equivalent to similar studies^43^. *T-*tests were conducted for all contrasts on each individual sample. Adjacent significant samples (p < 0.05) were clustered if the effects were significant on three or more neighboring channels. To control for multiple comparisons, a Monte Carlo estimate of the permutation p-value was calculated. Condition labels were randomly permuted (n = 1000), t-statistics were calculated and significant samples were clustered for each random partition. The proportion of randomly drawn partitions resulting in a larger test statistic (maximum sum of t-values over a cluster) than in real data gave the p-value. To control for testing both possible directions of an effect (A > B and A < B), clusters were considered significant at a Bonferroni corrected threshold of p = 0.05/2 = 0.025.

To assess the relationship between EEG spectral power and structural data (see method 2.6 and 2.7), the statistical group-level mask of the identified cluster from the comparison low vs. high load was used (see result section 3.2). Load-specific mean power values within the mask were extracted for each participant separately. Subjects with power values that differ more than three standard deviations from the mean would have been excluded from further analyses – however, no such outliers were detected.

### Image Acquisition, Processing and Statistical Analyses - VBM

Structural MRI data were measured using a 3T MR system (Siemens Trio) with a standard 32-channel head coil. First, whole-brain multiparameter mapping (MPM)^44^ was conducted on the basis of multi-echo 3D FLASH (fast low angle shot) images at 1 mm isotropic resolution with predominantly proton density (PD), magnetization transfer (MT) or T_1_ weighting. The total scanning time was approximately 20 minutes. Subsequently, diffusion-weighted images were acquired (see method 2.7).

The Statistical Parametric Mapping (SPM8) framework (Wellcome Trust Center for Neuroimaging, London) was used for data processing. The semi-quantitative parameter map of MT represents the percentage loss of magnetization induced by the MT saturation pulse and was calculated as described in^45,46^.

The MT maps where segmented into grey matter (GM), white matter (WhM) and cerebrospinal fluid (CSF) for voxel-based morphometry (VBM) within the unified segmentation approach^47,48^. MT maps were used for segmentation to help separating the effects of iron concentration from atrophy^49,50^. The resulting GM images were non-linearly transformed to standard MNI (Montreal Neurological Institute) space using the diffeomorphic registration algorithm (DARTEL) implemented in SPM8^51^, scaled by the Jacobian determinants of the deformation field and smoothed with an isotropic Gaussian Kernel of 6 mm full width at half maximum (FWHM).

For subsequent analyses in GM sub-space an explicit binary mask was used, generated as follows: Averages across all subjects for each tissue class (GM, WhM, CSF) were calculated using the Jacobian-modulated tissue probability maps in MNI space smoothed with a 3 mm Full Width Half Maximum (FWHM) isotropic kernel. Voxels were assigned to the tissue class for which their probability was maximal. If neither WhM nor GM probability exceeded 20%, the voxel was excluded from analysis. Only voxels within the GM mask were considered for analyses.

Whole-brain linear regression models as implemented in SPM8 were used to investigate the relationship between GM maps, and load-specific *d’*. Clusters with more than 25 voxel (k) and a p < 0.05 after family-wise error (FWE) correction at cluster-level^52^ were considered significant. The same applies to the regression models using the condition specific mean power values of the EEG analyses (see method section 2.5).

Mean GM values of significant clusters were extracted and entered into subsequent partial correlation analyses with IBM SPSS, controlling for age. Subjects with GM values that differ more than three standard deviations from the mean would have been excluded from further analyses – however, no such outliers were detected. Calculating the required correlation coefficient with a power of at least 0.8 for the given sample size using G*Power 3.1^53,54^ resulted in a minimum number of 0.478.

### Image Acquisition, Processing and Statistical Analyses - DTI

Diffusion-weighted data were acquired in the same session as the MPM by using echo planar imaging (64 × 2 mm thick axial slices, field of view 256 × 256 mm^2^, and voxel size of 2 × 2 × 2 mm). The diffusion weighting was isotropically distributed along 60 directions by using a b-value of 1000 s/mm^2^ (echo time of 85 ms, repetition time of 7.7 s). Additionally, 10 b0 reference images with no diffusion weighting were obtained after every 6th diffusion weighted image. In order to maximize the signal-to-noise-ratio, three repetitions of this scan sequence were taken per participant for subsequent averaging. The acquisition time was 28 minutes. Data were evaluated with FMRIB Software Library (FSL) version 5.0.9. Diffusion data were corrected for gradient induced stretches and shears, as well as for simple head motion using affine registration to the first volume. Next, distributions on diffusion parameters at each voxel were built up by Markov Chain Monte Carlo sampling to enable probabilistic tractography, i.e. connectivity distributions, from seed to target masks. Based on previous studies, we focused on the tractography of temporo-parietal, temporo-frontal and fronto-parietal tracts^24^.

**Figure 2:**
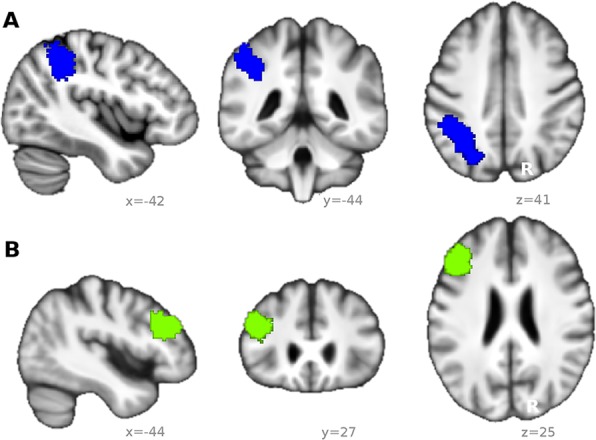
Masks used for DTI based tractography. Mask of the (**A**) left parietal cortex and (**B**) left dorsolateral prefrontal cortex (dlPFC). Both masks were based on a meta-analysis of 901 working memory studies (see text) and superimposed on a mean T_1_-weighted image derived from all subjects (for display purposes only).

Target masks (parietal cortex and dlPFC) were derived from a meta-analysis of 901 WM studies from the neurosynth-website^55^ (www.neurosynth.org, Jan 2016), thresholded at z = 3.09. For the MTL, the PHC cluster involved in WM as found in the VBM analysis was used as seed mask (see result section 3.3.1). Since this cluster lateralized to the left hemisphere, only left dlPFC and left parietal cortex were used as target masks (Fig. 2). The PHC mask (resulting from grey matter analysis) was dilated with a spherical kernel (radius 2 mm) to ensure inclusion of voxels containing white matter. Seed and target masks were transformed to individual diffusion space using warp fields derived from linear and non-linear registration with individual anatomical T1 images (skull-stripped in SPM). For the connection between dlPFC and parietal cortex, dlPFC was used as target and parietal cortex as seed.

From each of the voxels within the seed-mask, 5000 streamline samples were generated with a step length of 0.5 mm and a curvature threshold of 0.2 using probabilistic tractography as implemented in FSL. The number of samples passing through the voxels of the target mask for the individual given number of voxels in the seed mask was used as a measure of neuronal connectivity between the seed and target. Subjects with connectivity values that differ more than three standard deviations from the mean were excluded from further analyses. This was the case for two subjects in the data for the frontal-parietal tract and for one subject in the temporo-frontal tract.

The connectivity values were entered into a subsequent partial correlation analysis with *d’* and mean load-specific power values from the EEG data (see method section 2.5) using IBM SPSS. Correlations were controlled for participants’ age and size of the individual target masks. To account for multiple comparisons, correlations were considered to be significant at the Bonferroni corrected threshold of p = 0.05/4 = 0.0125 (medium and high load for *d’* and EEG data, respectively).

### Correction for multiple comparison

As described in the previous sections, corrections for multiple comparisons were applied. To summarize, in case of neuroimaging data (i.e. MRI and EEG), toolbox-specific inbuild functions were used, i.e. FWE-correction on cluster level in SPM^52^ for the VBM data or non-parametric cluster-based permutation test in fieldtrip for EEG^42^ (see 2.5 and 2.6). When data was further extracted for correlations, but also for the analyses of behavioral data, the alpha-level of 0.05 was divided by the number of possible tests to adjust the threshold (see each separate method section for details).

## Results

### Behavioral data

**Figure 3:**
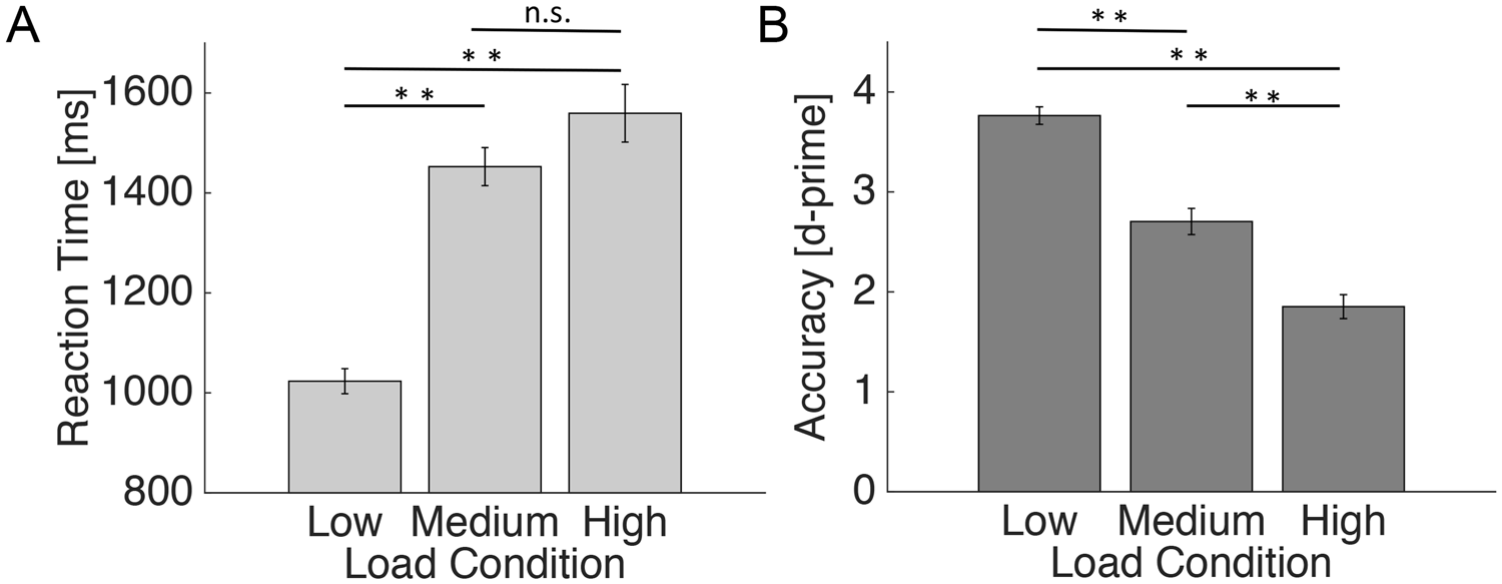
Behavioral results. (**A**) Reaction time increased and (**B**) accuracy decreased as a function of load. Average mean values are plotted with standard errors (** p < 0.001; n.s. = not significant, p > 0.017).

One-way ANOVAs revealed a highly significant (p < 0.001) effect of load (low, medium, high) on reaction time (RT; F (2, 90) = 44.7) and accuracy (*d’*; F (2, 90) = 70.5). As expected, accuracy decreased and RT increased as a function of load (Fig. 3). Post-hoc t-tests showed that performance under low load was significantly faster and more accurate than medium and high load (all p < 0.001); the comparison between medium and high load showed a significant difference for accuracy (p < 0.001), but (when Bonferroni-corrected) not RT (p = 0.03). Therefore, to simplify further analyses, only *d’* but not RT was used. Importantly, since the between-subject variability of *d’* in the low load condition was rather low (18 out of 31 subjects scoring more than 95% correct answers), all subsequent regression analyses with *d’* only included medium and high load.

### EEG data

**Figure 4:**
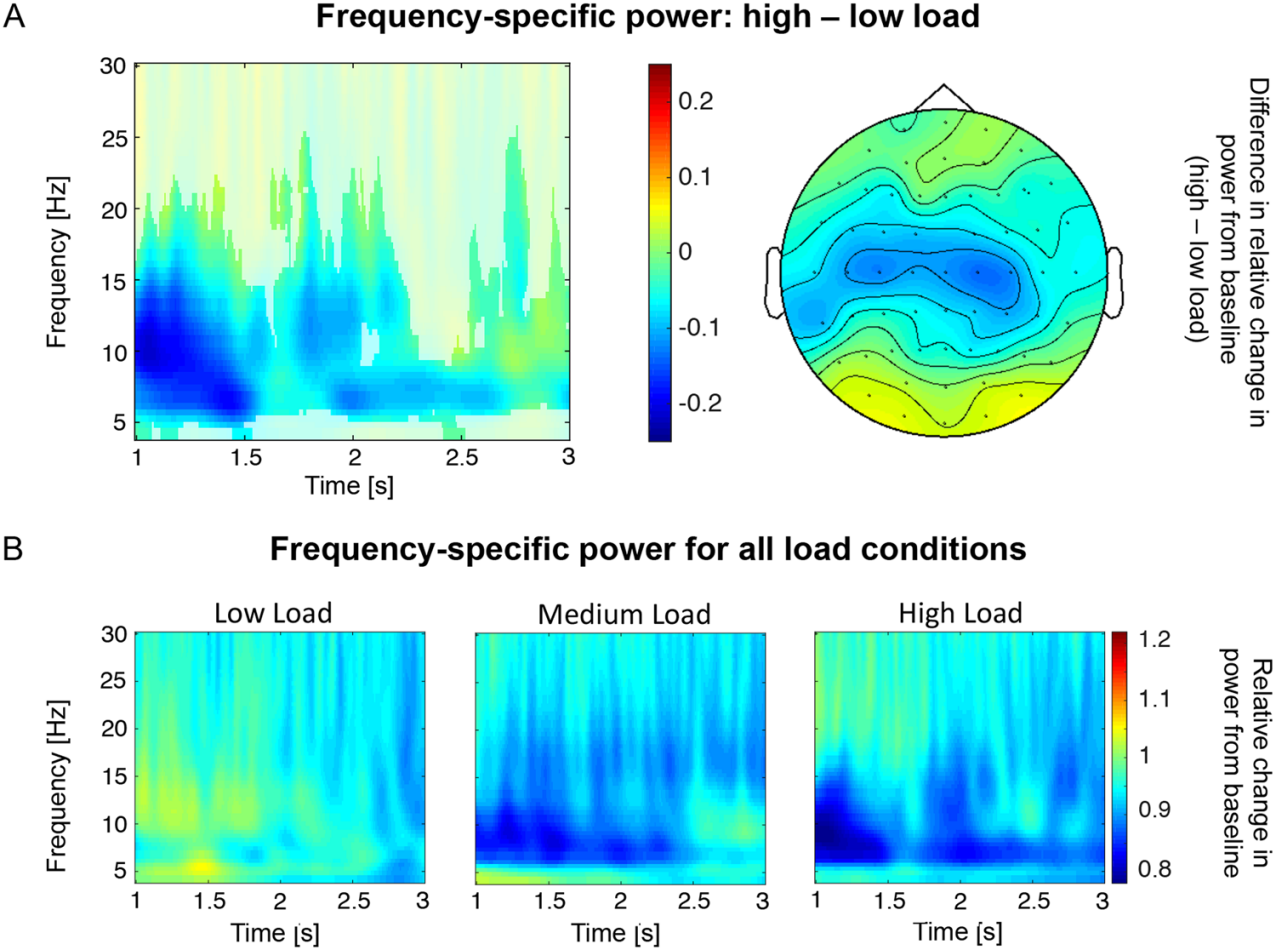
Neural oscillations during retention. (**A**) Theta-alpha power was significantly decreased during the retention period for high vs. low load (averaged over all electrodes). Non-significant samples are displayed opaque. The right plot shows the topographical distribution of the effect on the left, averaged over significant time windows and frequencies (all electrodes significant). (**B**) Time-frequency plots of the relative change in power from baseline for each load condition during the retention period (averaged over all electrodes).

The Monte Carlo cluster-based permutation test in 3D space (time, frequency, channel) revealed a significant difference (p = 0.001) between spectral power for low vs. high load. More specifically, we identified lower power from 4 to 25.5 Hz in the high vs. low load condition (Fig. 4A). The effect was most pronounced in the theta-alpha band (5–12 Hz) and broadly distributed across all scalp electrodes, with a focus on central electrodes. It was prominent throughout the entire analyzed retention phase (1–3 s after retention onset). Additionally, we identified a similar cluster (same channel and frequency distribution) with lower power for medium compared to low load (p = 0.004), spanning from 1 s until 2.17 s after retention onset (not shown). There was no significant negative cluster for the comparison high vs. medium load, i.e. no decrease in power. Finally, there was no effect in the other direction, i.e. no significant increase in power with load, in any comparison.

### Relationship between brain structure, behavior and neural oscillations

#### Gray matter volume

**Figure 5:**
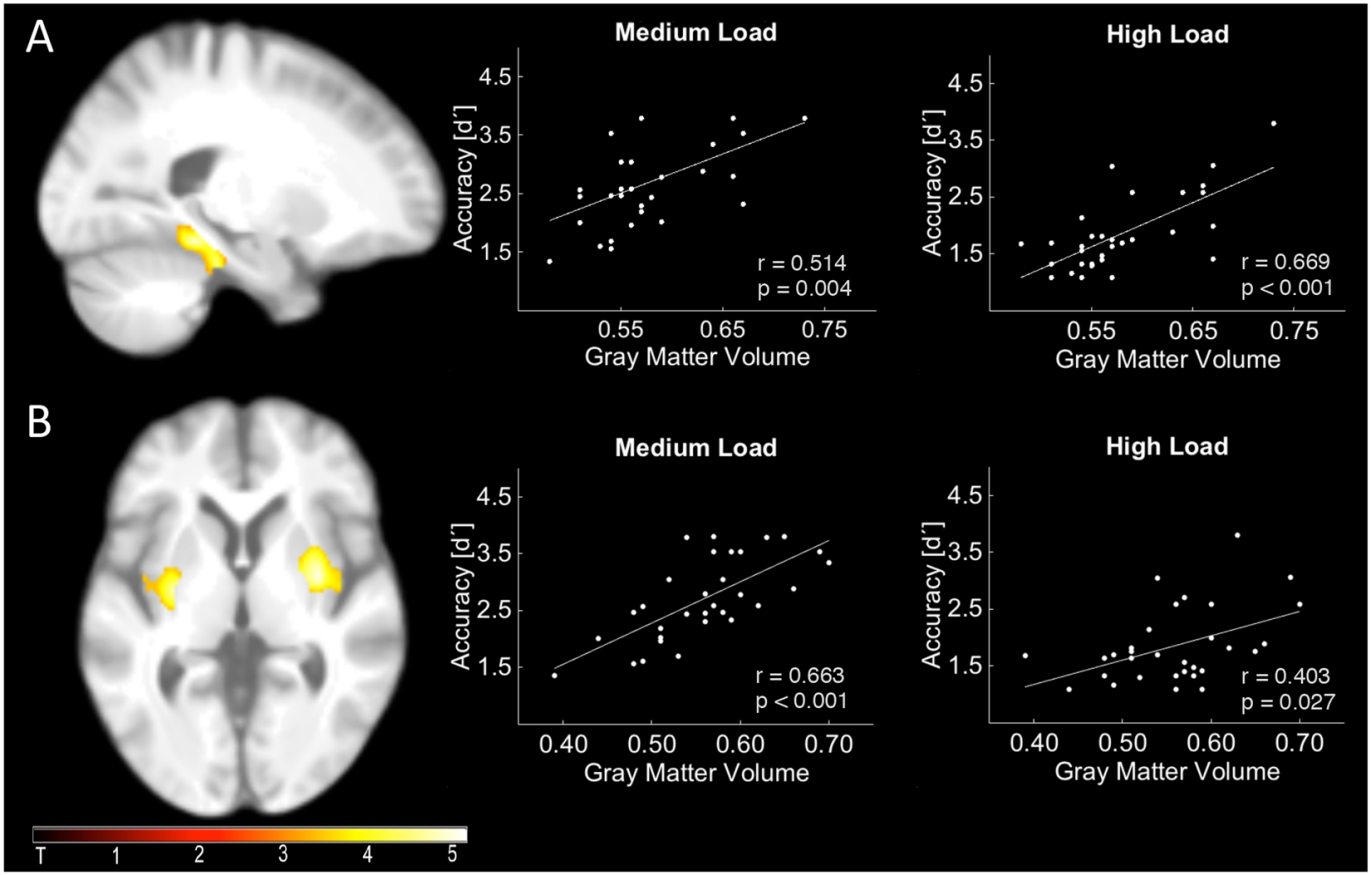
Relationship between gray matter and accuracy. (**A**) Within the parahippocampal cortex, gray matter volume correlated with high and (post-hoc) medium load. (**B**) Within the dorsal striatum (putamen/pallidum), gray matter volume correlated with accuracy under medium, with a trend for correlation with high load (post-hoc). Statistical parametric maps (SPM) in (**A**,**B**) (left column) were based on whole brain regression analyses with either high (**A**) or medium (**B**) load, and parameter estimates were extracted for planned post-hoc analysis with accuracy for the respective load condition (regression plots).

To examine the relationship between accuracy and structural integrity of gray matter (GM), load specific *d’* was used as a covariate in a whole brain linear regression model on grey matter maps. For high load, there was a positive correlation between accuracy and GM (p < 0.05, FWE-corrected) within the left PHC (MNI coordinates of the peak: x = −22, y = −37; z = −15; k = 546; Fig. 5A) and the supplementary motor area (MNI coordinates of the peak: x = 63, y = −9; z = 27; k = 495). Accuracy under medium load correlated significantly with voxels in two bilateral clusters centered at the dorsal striatum (i.e. putamen/pallidum; MNI coordinates of the peak; left: x = −28, y = −9; z = 3; k = 629; right: x = 31, y = 0; z = 4; k = 874; both p < 0.05, FWE-corrected; Fig. 5B), as well as the cerebellar lobule IX (MNI coordinates of the peak: x = −8, y = −54; z = −42; k = 766).

This FWE-corrected analysis suggests a clear distinction between load conditions and brain region (PHC associated with high load, and dorsal striatum associated with medium load). However, to fully characterize the relationship between structure and performance in each load condition, we extracted mean values from the identified FWE-corrected clusters. More precisely, GM values were extracted from the PHC (Fig. 5A) and bilateral dorsal striatal cluster (Fig. 5B) and entered into a partial correlation analysis with the condition specific *d’* values, controlling for age (Bonferroni corrected threshold of p = 0.05/2 = 0.025). It revealed a positive correlation between GM volume of the PHC and *d’* in the medium load condition (r = 0.514, p = 0.004), and, as shown in the whole-brain analyses, in the high load condition (r = 0.669, p < 0.001). A direct comparison of the correlations^56^ revealed no significant difference between medium and high load (p = 0.29, two-tailed).

For the dorsal striatum, a similar pattern emerged: apart from the positive correlation between gray matter and *d’* under medium load (r = 0.663, p < 0.001) as shown in the whole-brain analyses, a trend for the high load condition (r = 0.403, p = 0.027) has been found. Importantly, the direct comparison of both correlations revealed no significant difference between medium and high load (p = 0.08).

In a subsequent whole brain regression analysis, we investigated the relationship between GM volume and theta-alpha power as extracted from WM maintenance (see method section 2.6). It revealed no statistically significant effects for any load condition.

#### Connectivity strength

**Figure 6:**
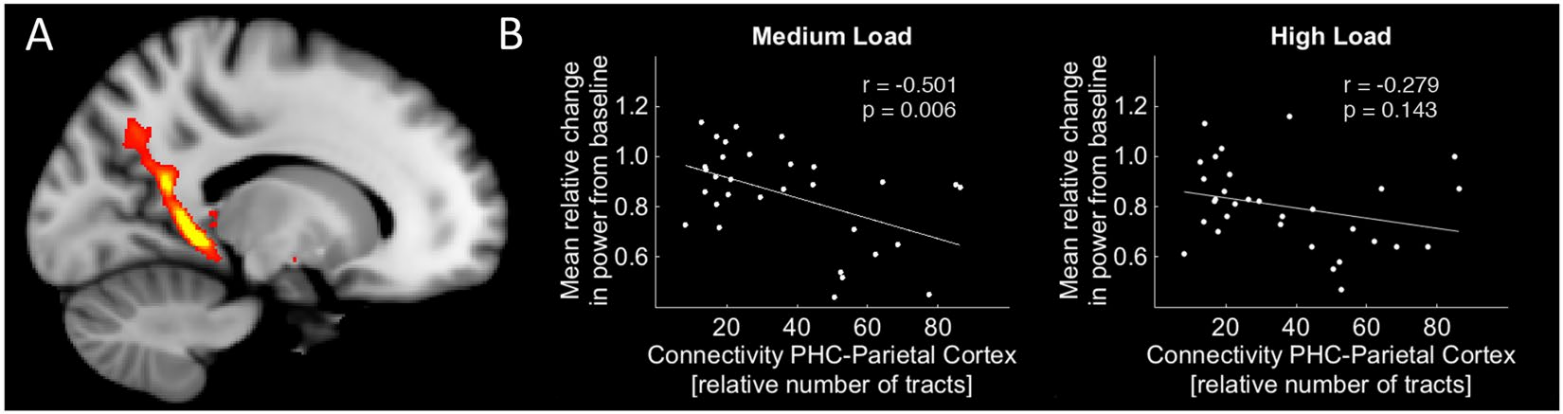
DTI tractography results. (**A**) Sample tract between parahippocampal cortex (PHC) and parietal cortex for one single subject (thresholded at 25% connectivity for display purposes). (**B**) Between-subjects correlations between connectivity strength and the load-specific power change as derived from EEG (see Fig. 4A).

Finally, we investigated white matter connectivity with a focus on temporo-parietal, temporo-frontal and fronto-parietal tracts, and their relation to behavioral performance and with WM-related EEG spectral power. To this end, the parahippocampal brain region identified above (Fig. 5A) was used as seed region; targets were frontal and parietal regions, respectively (see methods). In a next step, connectivity between frontal and parietal regions was calculated. Partial correlations (controlled for mask-size and age) of the EEG data and the connectivity values derived from DTI tractography revealed a negative association between PHC - parietal cortex connectivity (temporo-parietal tract; Fig. 6A) and the mean spectral power for the medium load condition (r = −0.501; p = 0.006) but not for the high load condition (p = 0.143; Fig. 6B). However, the direct comparison between correlations^56^ revealed no significant difference between both load conditions (p = 0.08).

There was no significant correlation between tract strength and accuracy in any tract under investigation (p > 0.0125; see Supplementary Table S1 for details).

## Discussion

We used EEG, MRI and DTI to investigate the structural and functional mechanisms underlying inter-individual differences in WM performance in healthy elderly. First, we found that behavioral accuracy decreased as a function of WM load, whereas RT showed the opposite pattern. Second, this was accompanied by modulations in the theta-alpha band that were directly related to tract strength between PHC and parietal cortex. More specifically, higher relative numbers of tracts were associated with stronger relative decreases in power from baseline (as expressed by a negative correlation). Finally, inter-individual differences in accuracy were predicted by gray matter volume of the PHC and dorsal striatum. As such, our data demonstrate a specific relationship between WM performance and structural integrity of the PHC and dorsal striatum in the healthy elderly. Moreover, they indicate that theta-alpha oscillations may serve as a mechanism to communicate between PHC and parietal cortex.

The increase in RT and decrease in accuracy with WM load at the behavioral level is in accordance with previous work^16,57^ and most likely reflects the different degrees of difficulty (Fig. 3). Importantly, theta-alpha power during retention also decreased with WM load (Fig. 4) suggesting a relationship between accuracy (*d’*) and neural oscillations. While this finding is at odds with previous studies reporting a load-dependent increase of theta and alpha power^15,16^, it is in line with a growing body of evidence suggesting a more complex picture. In fact, recent studies reported a power decrease in the theta-alpha band with WM load, which could be localized to occipital, occipitotemporal and parietal brain regions by using either source modeling^12,58^ or intracranial EEG^59^. Importantly, these power decreases were accompanied by power increases in fronto-medial networks^12,57,58^. However, such an additional effect could not be observed in our data, which may be due to our specific group of healthy elderly subjects. Indeed, McEvoy and colleagues found that, in contrast to young participants, elderly show a load-dependent decrease in power over parietal but also frontal regions and no evidence of power enhancement with increasing task demands, which may be due to neuronal compensatory strategies^17^. Finally, comparable to our results, several previous studies did not find differential effects for the theta and (low) alpha band, arguing against a clear functional dissociation during WM^12,13,59^. However, future studies are needed to address this open question.

A set of previous studies has shown a negative correlation between low-frequency oscillations and the blood oxygen level dependent (BOLD) signal as measured with fMRI^58,60–62^. This suggests that cortical event-related desynchronization (decrease of power) can be interpreted as a measure of neural activation^63,64^. In line with fMRI findings of a positive relationship between activity in parietal cortex and WM load^6,65^, it may indicate that our theta-alpha desynchronization reflects increased cortical activity. This resonates well with studies fitting load-dependent decreases of theta and (low) alpha power to the occipital, occipitotemporal and parietal regions^12,58,59^. As such, the parietal cortex may implement attentional control or store mental representations of visual scenes^5,6,65,66^ and insufficient engagement relates to erroneous maintenance of information^67^. Increased and desynchronized firing of parietal neurons in lower frequencies (<20 Hz) may further enable a neural representation that can drive activity in interconnected downstream brain regions such as the medial temporal lobe^68^ – an effect for which, to some degree indicated by our DTI results, the temporo-parietal tract may provide one possible structural basis.

Alternatively but not mutually exclusive, low frequency oscillations may provide a mechanism to allow long-range communication of segregated brain regions^69–71^. Our results from DTI tractography may further support this notion by showing that theta-alpha power in the medium load condition negatively correlated with tract strength between PHC and parietal cortex across subjects (Fig. 6). In other words, a stronger connectivity was associated with a stronger theta-alpha desynchronization (relative decrease in power from baseline). Although the precise mechanisms remain under debate^68,72,73^, this negative relationship might suggest inhibitory modulations between the parietal cortex and PHC during WM through theta-alpha oscillations, with the temporo-parietal tract as an anatomical pathway.

From a more general perspective, our findings show that the WM network is vulnerable to structural connectivity loss^74,75^, and more specifically they indicate that a low integrity of the temporo-parietal tract might results in impaired communication between the parietal cortex and PHC. Importantly, our analysis only included the left hemisphere (see method section 2.7). However, this was data driven and supposed to reduce analysis complexity instead of implying laterality. Indeed, previous studies show that structural integrity of both, left and right temporo-parietal tracts is associated with WM performance in the elderly^24^. Therefore, it is reasonable to assume the same underlying mechanisms (i.e. tract-desynchronization-interaction) also within the right hemisphere.

As another main finding, we can show that the integrity of the PHC and dorsal striatum (i.e. grey matter volume) explains behavioral variance in WM performance, which provides further evidence for a specific role of both structures in WM. Initial models have suggested a physiological and functional separation between WM and long-term memory^76,77^, but there is growing evidence that both memory systems closely interact^78^. Accordingly, parietal and prefrontal WM processes can be supported by the MTL if WM capacity is exceeded^7,8,79^. In this regard, the PHC may act as a buffer for WM representations by actively maintaining information during the retention period, especially for higher cognitive loads^80^. Our findings of a positive relationship between the PHC volume and WM performance are compatible with such a view and they indicate that higher integrity of the PHC contributes to better WM performance.

Similar to the PHC, the integrity of the dorsal striatum predicted inter-individual differences in WM performance (positive relationship), which is partly compatible with lesion-studies in patients^22^. However, it is likely that the dorsal striatum serves a different role than the PHC. Specifically, it has been argued that it provides a gating mechanism to control the flow of information to the PFC. In other words, the dorsal striatum may bias attention during encoding and maintenance towards relevant information^9,10^ to globally support WM processing^22^.

Interestingly, and contradictory to our initial hypotheses, we did not find any significant relationship between accuracy and dlPFC integrity or connectivity. Indeed, lesion studies in human patients^21,22^ and work in animals provide evidence for a link between age-related degeneration of the dlPFC and WM performance^81^. Since the PFC typically undergoes age related structural declines in humans, too^27^, a relationship to WM was expected. One possible explanation for our null finding is that structural alterations within the dlPFC may have been compensated by functional activity^82,83^. However, precise conclusions regarding the relationship between structural changes of the dlPFC and functional compensation need to be addressed in future work.

Finally, our design included three load conditions. On the one hand, as discussed above, there was a clear distinction between all three load conditions at the behavioral and oscillatory level, but low load *d’* could not be included in regression analyses due to low between subject variance (see results). Importantly, there were significant effects but no clear distinction between medium and high load accuracy (*d’*) in the PHC and dorsal striatum as shown by post hoc ROI analysis (Fig. 5). This suggests that both brain regions are equally involved in the processing of both WM conditions. The DTI analyses, on the other hand, is less clear (Fig. 6). Although a significant relationship between connectivity and EEG power could be observed in the medium but not high load condition, there was no significant difference between both correlations. In this regard, it is important to note that our results are based on a medium sample size (n = 31) and, as in most empirical studies, one cannot fully exclude the possibility of false-positives or false-negatives. However, to minimize erroneous findings, the data and analyses were controlled for outliers, based on adequate power and the alpha-levels were Bonferroni corrected. Nevertheless, validating our findings in future studies possibly with larger samples is desirable and may further clarify the relationship between the temporo-parietal tract and WM related theta-alpha oscillations.

Another limitation of our study is the absence of a group of younger subjects. While the inclusion of only healthy eldery was based on the rationale described above (see introduction), it is important to note that similar structure-behavior interactions could be present in younger subjects as well. However, we speculate that these interactions should be more pronounced during older age, due to the structural decline resulting in larger variability. Similar approaches have been used previously^24,29–31^ and in general, our results indicate that a degeneration of the structures discussed above relate to changes in neuronal activation and to the decline in WM performance during healthy aging. However, the direct influence of age-related changes may be tested in future studies that include a group of younger subjects or use a longitudinal design.

Finally, we would like to note that conclusions about neural activation specific to the retention phase can only be drawn from the EEG data, but not the VBM-behavior interactions. To address the question which specific aspect of WM processing (encoding, retention, retrieval) is affected by differences in structural integrity of the PHC and the striatum, future studies may combine fMRI and structural measures with paradigms testing WM.

To conclude, our findings shed new light on the structural and functional mechanisms underlying individual differences in WM functioning. We combined EEG, VBM and DTI in a group of healthy elderly, to provide new evidence that WM performance in the aging brain critically depends on the integrity of the PHC and striatal gray matter, while neural theta-alpha oscillations may provide a mechanism for communication between the nodes of the WM network.

## Electronic supplementary material

Supplementary Table S1

## Data Availability

The data that support the findings of this study are available on reasonable request from the corresponding authors (T.S. or N.B.). The data are not publicly available due to data security regulations by the local ethics committee.
