# Supplementary material for: Working memory performance in the elderly relates to theta-alpha oscillations and is predicted by parahippocampal and striatal integrity
Source: Sci Rep. 2019 Jan 24;9:706. doi: 10.1038/s41598-018-36793-3 (PMC6345832; doi:10.1038/s41598-018-36793-3)
Supplement: Supplementary file 1 — Supplementary Table S1 [file 41598_2018_36793_MOESM1_ESM.docx]

**Working memory performance in the elderly relates to theta-alpha oscillations and is predicted by parahippocampal and striatal integrity**

*Tineke K. Steiger ^1,2^, Nora A. Herweg ^2,3^, Mareike M. Menz ^2^, *Nico Bunzeck ^1,2^

(1) Institute of Psychology I, University of Luebeck, 23562 Luebeck, Germany

(2) Department of Systems Neuroscience, University Medical Center Hamburg-Eppendorf, 20246 Hamburg, Germany

(3) Department of Psychology, University of Pennsylvania, PA 19104, Philadelphia, USA

**Corresponding authors:*

Tineke Steiger and Nico Bunzeck

Institute of Psychology I, University of Luebeck

Ratzeburger Allee 160, 23562 Luebeck, Germany

Phone: +49-(0) 451 3101 3601

Email: tineke.steiger@uni-luebeck.de or nico.bunzeck@uni-luebeck.de

**Supplementary Table S1**: Correlation coefficients and significance-values (2-tailed) for the partial correlation analyses between working memory accuracy [*d'*] and connectivity [relative numbers of tracts], corrected for age. Different sample sizes (n) were due to outlier correction (see method section 2.7). Correlations were considered to be significant at a Bonferroni corrected threshold of p = 0.0125 (see method section 2.7).

|  | Temporo-Parietal (n=31) | | | Temporo-Frontal (n=30) | Fronto-Parietal (n=29) | |  |
| --- | --- | --- | --- | --- | --- | --- | --- |
| *Medium Load* |  |  | | | |  | |
| Correlation (r) | 0.243 | | -0.081 | | | 0.191 | |
| Significance (p) | 0.195 | | 0.678 | | | 0.330 | |
| *High Load* |  | |  | | |  | |
| Correlation (r) | -0.085 | | 0.060 | | | -0.051 | |
| Significance (p) | 0.655 | | 0.758 | | | 0.795 | |
